# Second edition of 'The Bethesda System for reporting cervical cytology' – atlas, website, and Bethesda interobserver reproducibility project

**DOI:** 10.1186/1742-6413-1-4

**Published:** 2004-10-21

**Authors:** Ritu Nayar, Diane Solomon

**Affiliations:** 1 Northwestern University Feinberg School of Medicine, Feinberg 7-210, 251 East Huron Street, Chicago IL 60611, USA; 2 Division of Cancer Prevention, NCI, NIH, DHHS, 9000 Rockville Pike, Bethesda, MD 20892, USA

## Abstract

A joint task force of the American Society of Cytopathology (ASC) and the National Cancer Institute (NCI) recently completed a 2-year effort to revise the Bethesda System "blue book" atlas and develop a complementary web-based collection of cervical cytology images. The web-based collection of images is housed on the ASC website, which went live on November 5^th^, 2003; it can be directly accessed at .

The second edition of the Bethesda 'blue book' atlas maintains an easy to read format with bulleted morphologic criteria and half-page color illustrations. The content has been divided into chapters based on the major 2001 Bethesda System interpretive categories. Highlights of the new edition include: (1) incorporation of liquid based cytomorphologic criteria and images; (2) new sections addressing ancillary testing, educational notes and recommendations, computer assisted interpretation and anal-rectal cytology; and (3) inclusion of sample reports, references, and detailed legends for images. Overall, the second edition has tripled in size from the original version, with a total of 186 images of which 90% are new images and 40% are from liquid based specimens. Some are classic examples of an entity while others have been selected to illustrate interpretive dilemmas or "borderline" cytomorphologic features that may not be interpreted in the same way by all cytologists.

In parallel with production of the Bethesda book atlas, the ASC-NCI task force has also developed a Bethesda web-based collection of images. Approximately 350 images, (40% of which are from liquid based specimens) along with linked explanatory notes, including all the images in the published atlas, can be viewed on this site. The website is user friendly and has several search modalities for viewing the images including searching by Bethesda terminology, atlas chapter headings, keyword(s), or preparation type. It also allows for individual self-assessment by participating in a "self test" in which viewers can compare their interpretation to other participants' responses.

The image selection process for the book atlas and website involved a multistage review: *Step 1*: individual Bethesda forum group members *(32 participants) *reviewed and selected images for their chapter from among those in the first edition of the atlas and new submissions; and *Step 2*: the images selected from Step 1 were reviewed individually ("validated") by 13 task force members and scored on a scale of 1–5 for agreement with interpretation and quality of image. In all over 1000 images were reviewed of which 186 images were selected for the atlas and an additional 163 for the website.

A subset (n = 77) of the book atlas images were posted as "unknowns" on the Internet from mid July to mid September 2003 as part of a study – the Bethesda Interobserver Reproducibility Project (BIRP). The site was open to the cytopathology community to view the images and provide their interpretations. Immediately after submitting their response, participants were able to view a histogram of the distribution of results submitted by all prior participants for that image. Over 600 cytologists from around the world participated in BIRP. Summary histograms for each of the 77 images can be viewed on the Bethesda atlas website (select BIRP images from the left menu). Preliminary BIRP results presented at the ASC annual meeting in Orlando in November 2003 showed that Negative for Intraepithelial Lesion or Malignancy (NILM) and Low Grade Squamous Intraepithelial Lesion (LSIL) reference images attained the highest concordance scores, while glandular abnormalities demonstrated the most splay in distribution of interpretations. BIRP analyses are ongoing and further results should be available in 2004.

## ASC-NCI Working Group for the Second Edition Bethesda Atlas and Website

***ASC Bethesda 2001 Task Force:****Ritu Nayar *(Chair), Diane Solomon (Co-Chair, NCI) *George Birdsong *(Adequacy), *Jamie Covell *(Glandular Lesions), *Ann Moriarty *(Endometrial cells), *Dennis O'Connor *(Educational notes and recommendations), *Marianne Prey *(Computer assisted interpretation), *Steve Raab *(Ancillary testing), *Mark Sherman *(Atypical squamous cells), *Sana Tabbara *(Other malignant neoplasms), *Tom Wright *(Squamous lesions), *Nancy Young *(Non-neoplastic findings).

*ASC Consultants: *ASC 2002/2003 Presidents: *Diane Davey and Dave Wilbur*.

*Information Technology representatives: Mike Montgomery (NCI) and Brandon Winbush (Northwestern University), Aquilent (Laurel, MD)*.

The second edition of the 'blue book' atlas can be ordered through Springer-Verlag (1-800-SPRINGE) for $34.95.

